# Expression of cotton PLATZ1 in transgenic *Arabidopsis* reduces sensitivity to osmotic and salt stress for germination and seedling establishment associated with modification of the abscisic acid, gibberellin, and ethylene signalling pathways

**DOI:** 10.1186/s12870-018-1416-0

**Published:** 2018-10-04

**Authors:** Shicai Zhang, Rui Yang, Yanqing Huo, Shasha Liu, Guodong Yang, Jinguang Huang, Chengchao Zheng, Changai Wu

**Affiliations:** State Key Laboratory of Crop Biology, College of Life Sciences, Shandong Agricultural University, Tai’an, Shandong People’s Republic of China

**Keywords:** *Gossypium hirtusum* L, PLATZ, Abiotic stresses, Ethylene, ABA, GAs

## Abstract

**Background:**

Zinc-finger transcription factors play central roles in plant growth, development and abiotic stress responses. PLATZ encodes a class of plant-specific zinc-finger transcription factor. However, biological functions or physiological mechanism controlled by PLATZ are currently limited.

**Results:**

*GhPLATZ1* transcripts were considerably up-regulated by NaCl, mannitol, abscisic acid (ABA) and gibberellin (GA) treatments. Transgenic *Arabidopsis* by ectopic expression of *GhPLATZ1* exhibited faster seed germination and higher seedling establishment under salt and mannitol stresses than those of wild type (WT), indicating enhanced osmotic insensitivity in *GhPLATZ1* transgenic *Arabidopsis*. The ABA content in dry seeds of *GhPLATZ1* transgenic *Arabidopsis* was lower than that of WT whereas the ABA content was not changed in germinating seeds under salt stress. Seed germination was faster than but the seedling establishment of transgenic *Arabidopsis* was similar to WT. Besides, *GhPLATZ1* transgenic and WT *Arabidopsis* exhibited insensitivity to paclobutrazol (PAC), a GA biosynthesis inhibitor, whereas exogenous GA could eliminate the growth difference between *GhPLATZ1* transgenic and WT *Arabidopsis* under salt stress. Moreover, exogenous 1-aminocyclopropane-1-carboxylic acid (ACC), an ethylene precursor, exerted similar effects to GA*.* Furthermore, *ABI4* and *ETO1* transcripts were significantly down-regulated, whereas *ACS8* was up-regulated in *GhPLATZ1* transgenic *Arabidopsis* under salt stress.

**Conclusions:**

In conclusion, GhPLATZ1 had broad influence in responses to salt and mannitol stresses in transgenic *Arabidopsis* during seed germination and seedling establishment. The effect of GhPLATZ1 expression in transgenic Arabidopsis might be mediated by the ABA, GA, and ethylene pathways. Thus, this study provided new insights into the regulatory network in response to abiotic stresses in plants.

**Electronic supplementary material:**

The online version of this article (10.1186/s12870-018-1416-0) contains supplementary material, which is available to authorized users.

## Background

Impaired germination and postgermination growth are common among seeds exposed to salt and drought stresses. Plants can regulate and coordinate both growth and/or stress sensitivity to promote survival or escape from environmental stress by modifying the production, distribution, or signal transduction of hormones. Thus, identification of components involved in hormonal regulation during germination under abiotic stresses is essential.

A large number of genes encoding DNA-binding proteins, which play important roles in hormone mediated responses to abiotic stresses were identified in plants. For example, the major downstream components of abscisic acid (ABA) signaling, namely, ABA insensitive 3 (ABI3) [1], ABI4 [2] and ABI5 [3, 4], are positive regulators of ABA, and they are crucial in seed maturation, seed germination and seedling growth [5]. Fuctional loss of these genes causes fast germination under ABA and abiotic stresses. The GA signaling pathways depend on DELLA proteins including GA insensitive ones (GAI), repressor of *ga1–3* (RGA), repressor of *ga1–3*-like (RGL) 1, RGL2 and RGL3 [6, 7]. GA destabilizes the DELLA proteins, which act as growth repressors, by targeting ubiquitination and degradation [8]. EIN2 plays a key role in ethylene signaling and losing its function results in a hypersensitivity to salt and osmotic stress during germination and early seedling development in *Arabidopsis* [9]. *ERF* genes might also play a pivotal role in ethylene responsiveness and germination regulation in tomato (*Solanum lycopersicon*); *SlERF2* overexpression in transgenic lines causes premature seed germination [10].

Zinc-finger transcription factors are a relatively large family of plant transcription factors (approximately 15% of the total) and play central roles in plant growth and development [11, 12]. PLATZ1 was first isolated from pea [13]. PLATZ1 and its paralogs from *Arabidopsis* and other species have two consensus signatures, namely, C-x_2_-H-x_11_-C-x_2_-C-x_(4–5)_-C-x_2_-C-x_(3–7)_-H-x_2_-H (CHC_4_H_2_) and C-x_2_-C-x_(10–11)_-C-x_3_-C. However, the two consensus signatures are different from those of previously characterized zinc-binding motifs, such as RING (C_3_HC_4_), LIM (C_2_HC_5_) [14], GATA finger (C-x_2_-C-x_(17–18)_-C-x_2_-C (C_2_C_2_)) [15, 16], COSTANS/CONSTANS-like (CO/COLs) [17], and the DNA-binding one finger (Dof) [18]. Hence PLATZs encode a novel class of plant-specific zinc-finger transcription factors. Recently, Arabidopsis PLATZ1 and 2 were reported to positively regulate acquisition of dessication tolerance in seeds and vegetative tissues [19]. Maize PLATZ protein FL3 is involved in tRNA and 5S rRNA transcription through interaction with RNA polymerase III [20]. Multiple members of *PLATZ* genes exist in plants. For example, 12 *PLATZ* genes exist in the *Arabidopsis* genome (https://www.arabidopsis.org/servlets/Search?type=general&search_action=detail&method=1&show_obsolete=F&name=PLATZ&sub_type=gene&SEARCH_EXACT=4&SEARCH_CONTAINS=1/). Therefore, the roles of PLATZs remain largely unclear.

Cotton (*Gossypium hirsutum*) is one of the most important fibre and oil crops. Cotton seed germination and seedling establishment are severely impaired in abiotic stress conditions. The biological significance of cotton PLATZs has not yet been described. In this study, the first PLATZ form cotton, named as GhPLATZ1, was isolated and characterized. Our results indicated that the expression of *GhPLATZ1* was induced by abiotic and hormone stimuli. Ectopic expression of *GhPLATZ1* in *Arabidopsis* resulted in enhanced insensitivity to osmotic stresses, ABA and PAC. We deduced that GhPLATZ1 might play important roles in regulating hormone-mediated osmotic stress during cotton seed germination and seedling establishment.

## Results

### Characterization of GhPLATZ1Transcription factor

We previously showed that a gene (GenBank ID: JQ837703) from cotton was significantly induced by salt stress [21]. The deduced amino acid sequence was homologous to PLATZ1 from peas and PLATZs from *Arabidopsis*. Given that the gene was the first isolated PLATZ from cotton, we named it as *GhPLATZ1*. Genome-wide searching in *G.hirtusium* and *Arabidopsis thaliana* revealed that 9 *PLATZ* genes exist in cotton (https://phytozome.jgi.doe.gov) and 12 are in *Arabidopsis* [the Arabidopsis Information Resource (TAIR), https://www.arabidopsis.org/servlets/Search?type=general&search_action=detail&method=1&show_obsolete=F&name=PLATZ&sub_type=gene&SEARCH_EXACT=4&SEARCH_CONTAINS=1]. GhPLATZs showed high identity with each other (≥79.56%) (Additional file 1: Figure S1A), and all of them shared numerous similarities to a subfamily of AtPLATZs, including AtPLATZ3, AtPLATZ11, and AtPLATZ12 (Additional file 1: Figure S1B). However, the roles of GhPLATZs remain unclear.

**Fig. 1:**
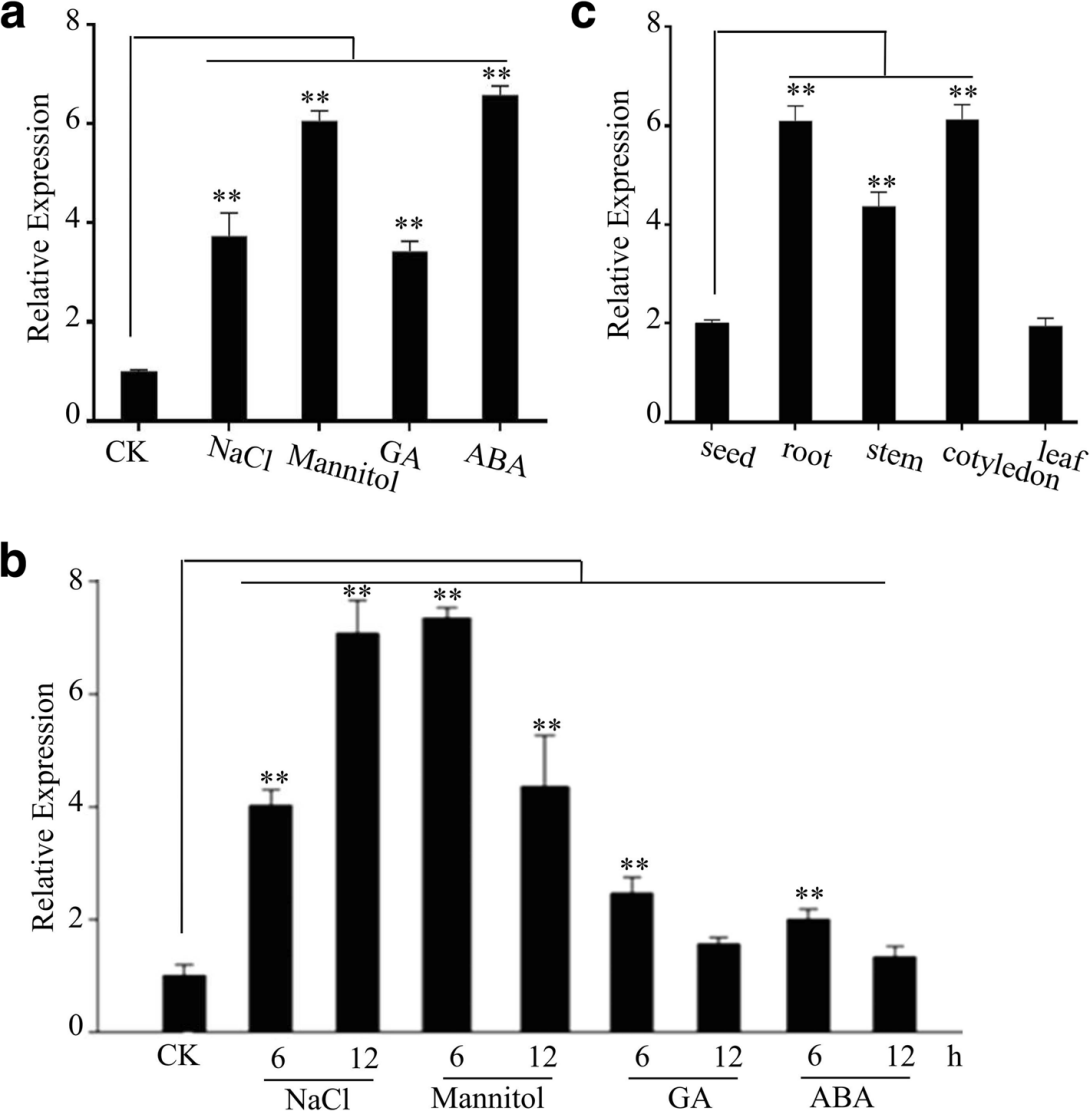
The expression patterns of *GhPLATZ1* analyzed by qRT-PCR. **a** *GhPLATZ1* expression in 20-day-old seedlings treated with or without 200 mM NaCl, 300 mM mannitol, 50 μM GA or 50 μM ABA for 6 h. **b** *GhPLATZ1* expression in 2.5-day germinated seeds in Hoagland solutions with or without 200 mM NaCl, 300 mM mannitol, 50 μM GA or 50 μM ABA for 6 and 12 h. The transcript levels were normalized to that of *GhUBI* (EU604080). Values are means ± SD of three replicates. **c** *GhPLATZ1* expression in seeds, roots, stems, cotyledens or leaves of 20-day-old cotton seedlings. At least three independent experiments were conducted

To explore the possible roles of GhPLATZ1 under salt stress and other abiotic stresses, the expression of *GhPLATZ1* was determined in 20-day-old seedlings by qRT-PCR using *GhUBI* (EU604080) as the reference gene. *GhPLATZ1* transcripts were induced by 3.8, 6.2, 6.8, or 3.6-fold by NaCl, mannitol, GA, or ABA treatment, respectively (Fig. 1a). During seed germination, *GhPLATZ1* transcripts were up-regulated by 4.1 and 7.2-fold or 7.5 and 4.5-fold by NaCl or mannitol treatment at 6 and 12 h, respectively, or two fold by GA and ABA treatments at 6 h (Fig. 1b). In addition, high levels of *GhPLATZ1* transcripts were detected in roots, stems and cotyledons, whereas low transcripts were found in leaves and seeds (Fig. 1c). These data suggested that *GhPLATZ1*may be involved in plant responses to environmental stimuli.

### *GhPLATZ1* transgenic *Arabidopsis* enhances insensitivity to osmotic stresses

**Fig. 2:**
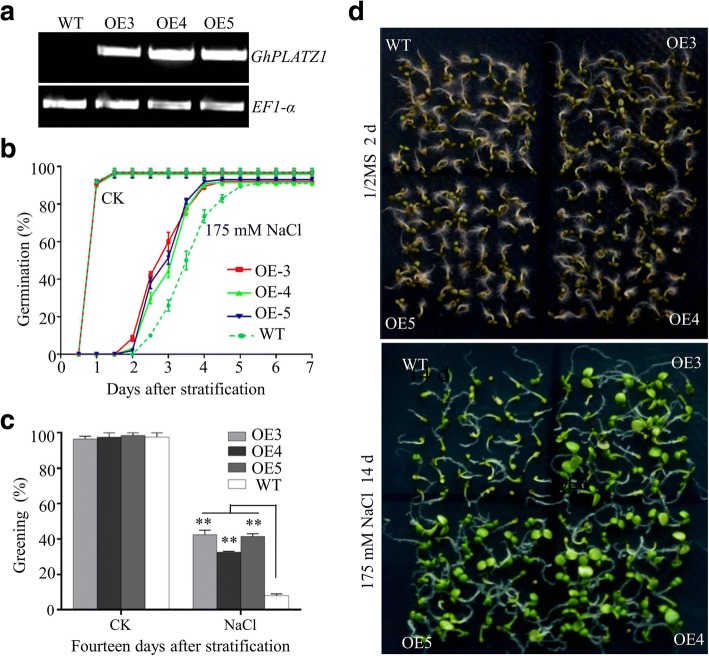
Phenotypic analysis of *GhPLATZ1* transgenic *Arabidopsis* under salt stress. **a** Determination of *GhPLATZ1* expression in the three independent transgenic T_3_ *Arabidopsis* homozygous OE3, OE4, and OE5 and WT. **b** Germination rates of WT, OE3, OE4, and OE5 seeds germinated on 1/2 MS medium with or without 175 mM NaCl for indicated time points after stratification. **c** Cotyledon greening of WT, OE3, OE4, and OE5 plants grown on 1/2 MS medium with or without 175 mM NaCl for 14 days after stratification. **d** Photographs of 2-day germinated seeds under 1/2 MS medium (upper) and 14-day-old seedlings under 1/2 MS medium containing 175 mM NaCl (lower). 1/2 MS medium was used as control (CK). Data showed the mean ± SD (*n* = 50) of three replicates

To further analyze the function of *GhPLATZ1*, we introduced 35S::*GhPLATZ1* into *Arabidopsis*, and obtained three independent transgenic lines with high levels of *GhPLATZ1* transcripts (Fig. 2a) for further analysis.

**Fig. 3:**
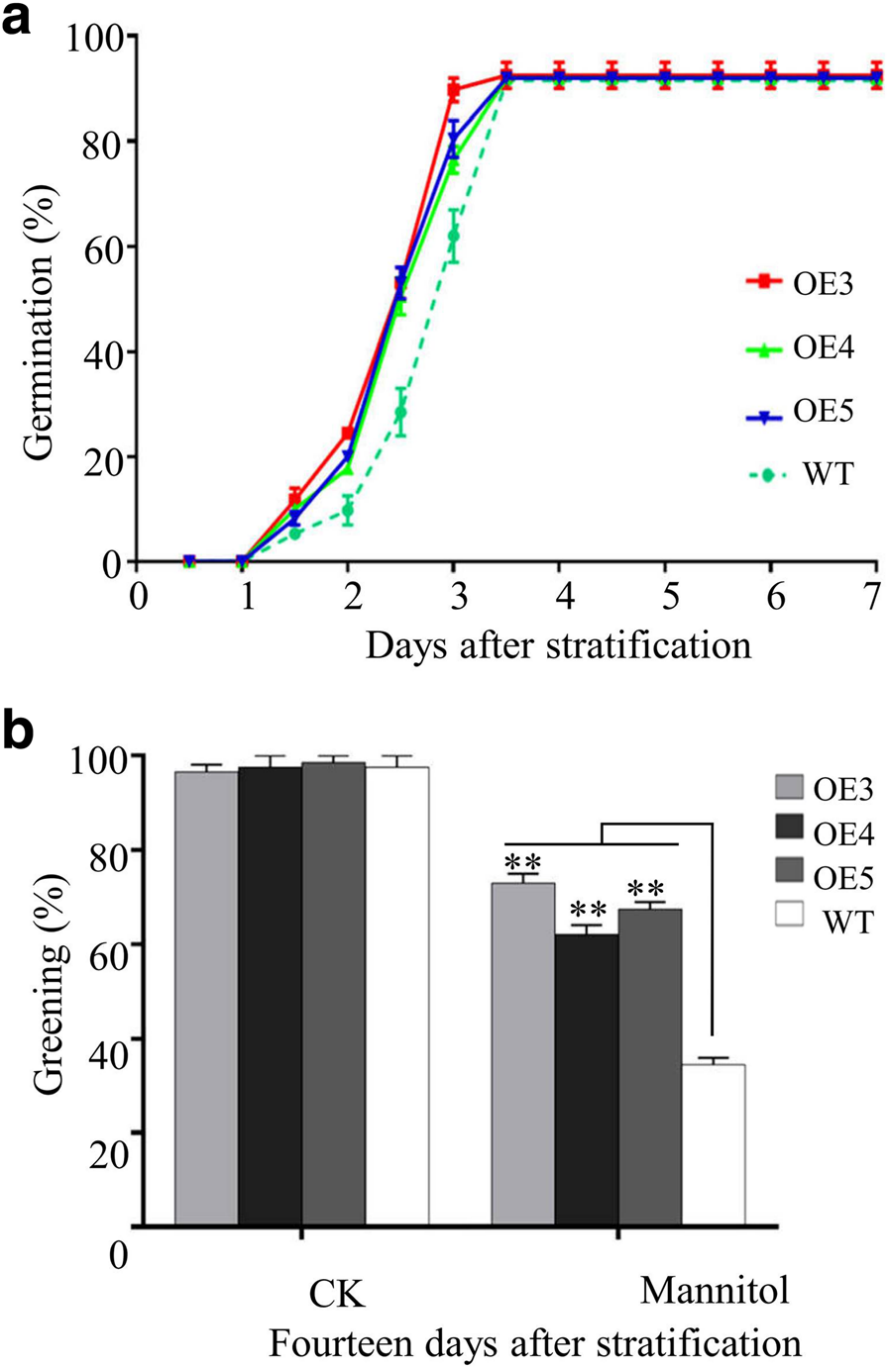
Phenotypic analysis of *GhPLATZ1* in transgenic Arabidopsis under mannitol stress. **a** Germination rates of WT, OE3, OE4, and OE5 seeds on 1/2 MS medium with 300 mM mannitol for indicated times. **b** Cotyledon-greening of WT, OE3, OE4, and OE5 plants grown on 1/2 MS medium with or without 300 mM mannitol for 14 days. Data show the mean ± SD (*n* = 40) of three replicates. At least 40 seeds per genotype were measured in each replicate

When seeds of WT and three transgenic lines were sown on 1/2 MS medium, the three transgenic lines showed similar seed germination and seedling establishment to WT did (Fig. 2b and c; Additional file 1: Figures. S2 and S3). However, when subjected to 1/2 MS medium with 175 mM NaCl, the three transgenic lines exhibited more than 20% higher germination rates than WT during 2.5–4 days, and reached the maximum germination rate 1.5 days earlier than WT (Fig. 2b). Cotyledon greening was also more than 20% higher in the transgenic lines compared to WT (Fig. 2c and d). Similar results were obtained when seeds of WT and three transgenic lines were subjected to 1/2 MS medium with 150 and 200 mM NaCl (Additional file 1: Figures. S2 and S3). Under treatment of 175 mM KCl, 100 mM Na_2_SO_4_, and 300 mM mannitol, the three transgenic lines also exhibited quicker germination than WT (Additional file 1: Figures S4A and S4B; Fig. 3a), and more than 30% higher cotyledon greening at 14 days for mannitol treatment (Fig. 3b) than WT. However, when treated with 15 mM LiCl, the three transgenic lines exhibited similar seed germination and cotyledon greening to WT (Additional file 1: Figure S4C and S4D). In order to determine whether ectopic expression of GhPLATZ1 alters the expression of the native PLATZ genes in *Arabidopsis*, the expression levels of 12 members of *AtPLATZ* genes were determined by qRT-PCR. Eleven of the 12 *AtPLATZ* genes were detected, and their expression levels were not significantly changed in *GhPLATZ1* transgenic lines (Additional file 1: Figure S5). However, we did not find a phenotypic difference between *GhPLATZ1* transgenic and WT *Arabidopsis* in vegetative tissues in the presence and absence of salt and mannitol stresses (data not shown). Thus, *GhPLATZ1* transgenic lines decreased the sensitivity to osmotic stress during seed germination and postgermination growth.

### GhPLATZ1 inhibits ABA biosynthesis

**Fig. 4:**
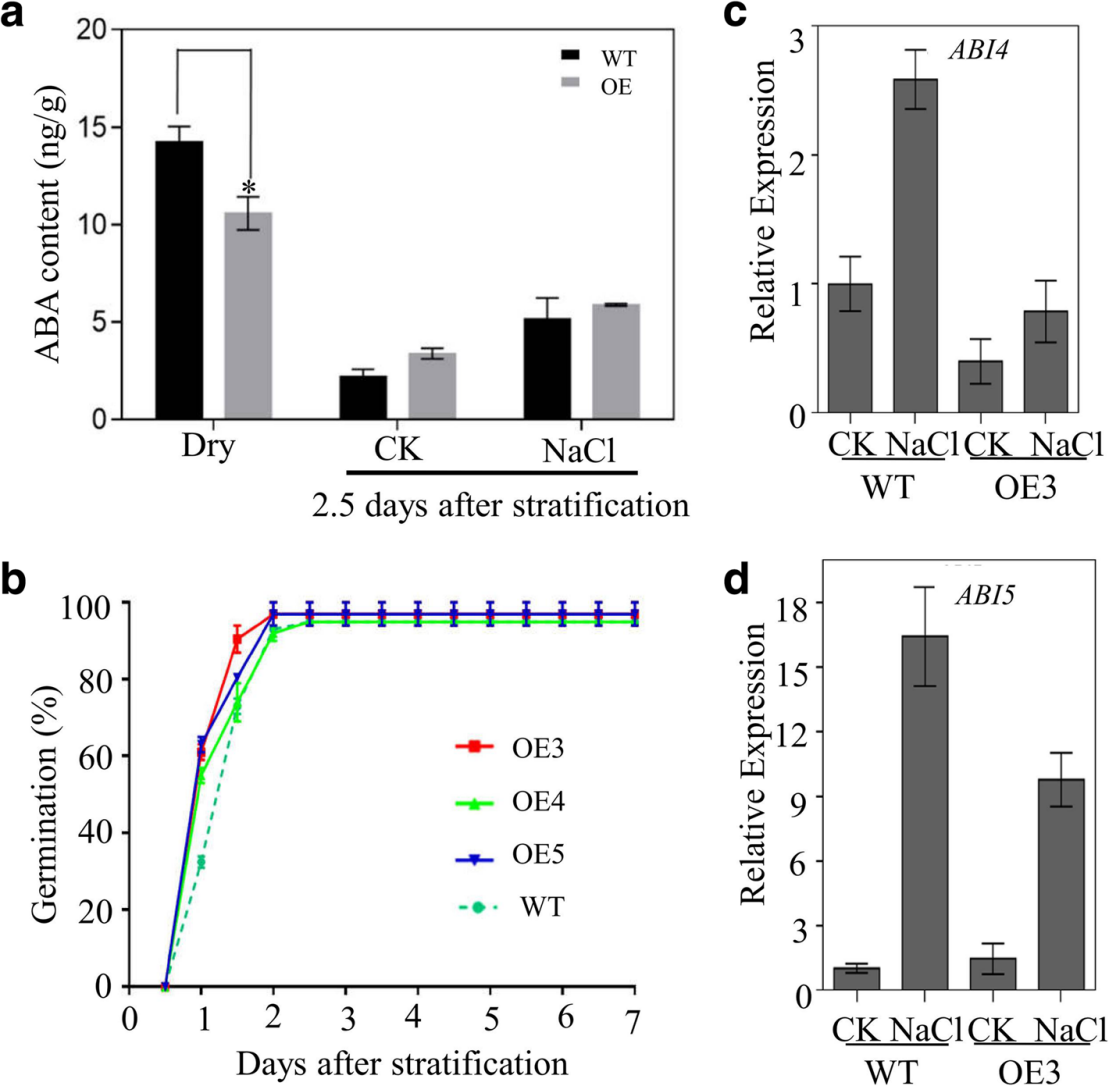
ABA response of GhPLATZ1 transgenic and WT plants. **a** Quantification of ABA content in dry seeds and 2.5-day germinated seeds in 1/2 MS medium with or without 175 mM NaCl. **b** Germination rates of WT, OE3, OE4, and OE5 seeds on 1/2 MS medium containing 5 μM ABA or indicated times. **c** The relative expression of *ABI4* and **d** *ABI5* in WT and OE3 seeds germinated for 2 days on 1/2 MS medium with or without 175 mM NaCl. Data show the mean ± SD (*n* = 49) of three replicates. At least three independent experiments were conducted

To investigate whether GhPLATZ1 functions through ABA in transgenic plants, ABA contents were measured. The result showed that dry seeds of *GhPLATZ1* transgenic lines contained significantly lower ABA level than WT, whereas germinated seeds of *GhPLATZ1* transgenic lines contained similar ABA level to WT at 2.5-day-germination stage with or without NaCl treatment (Fig. 4a). *GhPLATZ1* transgenic lines showed quicker germination than WT only before 2 d of germination in the presence of exogenous ABA, but similar phenotype after 2 d of germination (Fig. 4b). *ABI4* expression in *GhPLATZ1* transgenic lines was down-regulated 2-fold compared with WT at 2.5-day-germination stage with or without NaCl treatment (Fig. 4c); and *ABI5* expression was suppressed by less than two fold (Fig. 4d). These data indicated that GhPLATZ1 inhibited ABA biosynthesis in developing seeds, which might lead to rapid germination in the early stages.

### GhPLATZ1 involves GA and ethylene-mediated salt stress responses

**Fig. 5:**
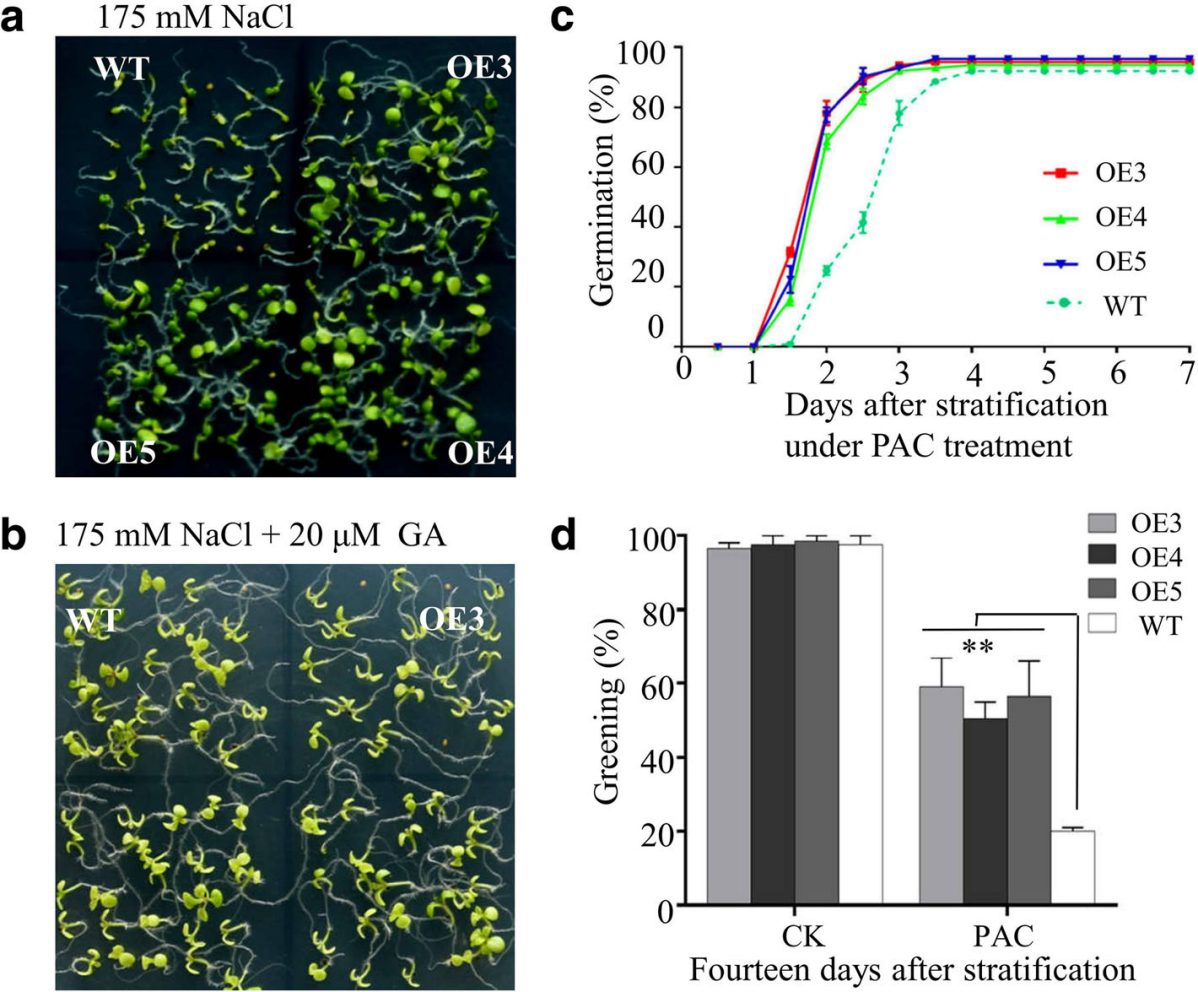
Responses of GhPLATZ1 transgenic and WT plants to GA and PAC. **a** Photograph of seedlings grown for 14 days on 1/2 MS medium containing 175 mM NaCl (**b**) Photograph of seedlings grown for 7 days on 1/2 MS medium containing 175 mM NaCl and 20 μM GA. **c** Germination rates of WT, OE3, OE4, and OE5 seeds on 1/2 MS medium containing 40 μM PAC for indicated times. Data show the mean ± SD (*n* = 56) of three replicates. **d** Cotyledon-greening of WT, OE3, OE4, and OE5 plants grown for 14 days on 1/2 MS medium with or without 40 μM PAC. Data show the mean ± SD (n = 56) of three replicates. At least three independent experiments were conducted

Upon comparing Fig. 5b with Fig. 5a, we found that seed germination was less sensitive to NaCl after the addition of exogenous GA in both *GhPLATZ1* transgenic lines and WT. Under PAC treatment, we observed 40% lower seed germination at 2–3 days (Fig. 5c), and 30% lower cotyledon greening at 14 days (Fig. 5d) in WT than in *GhPLATZ1* transgenic lines were obtained. Therefore, GhPLATZ1 was involved in the GA-mediated salt stress response of plants during seed germination and postgermination growth.

**Fig. 6:**
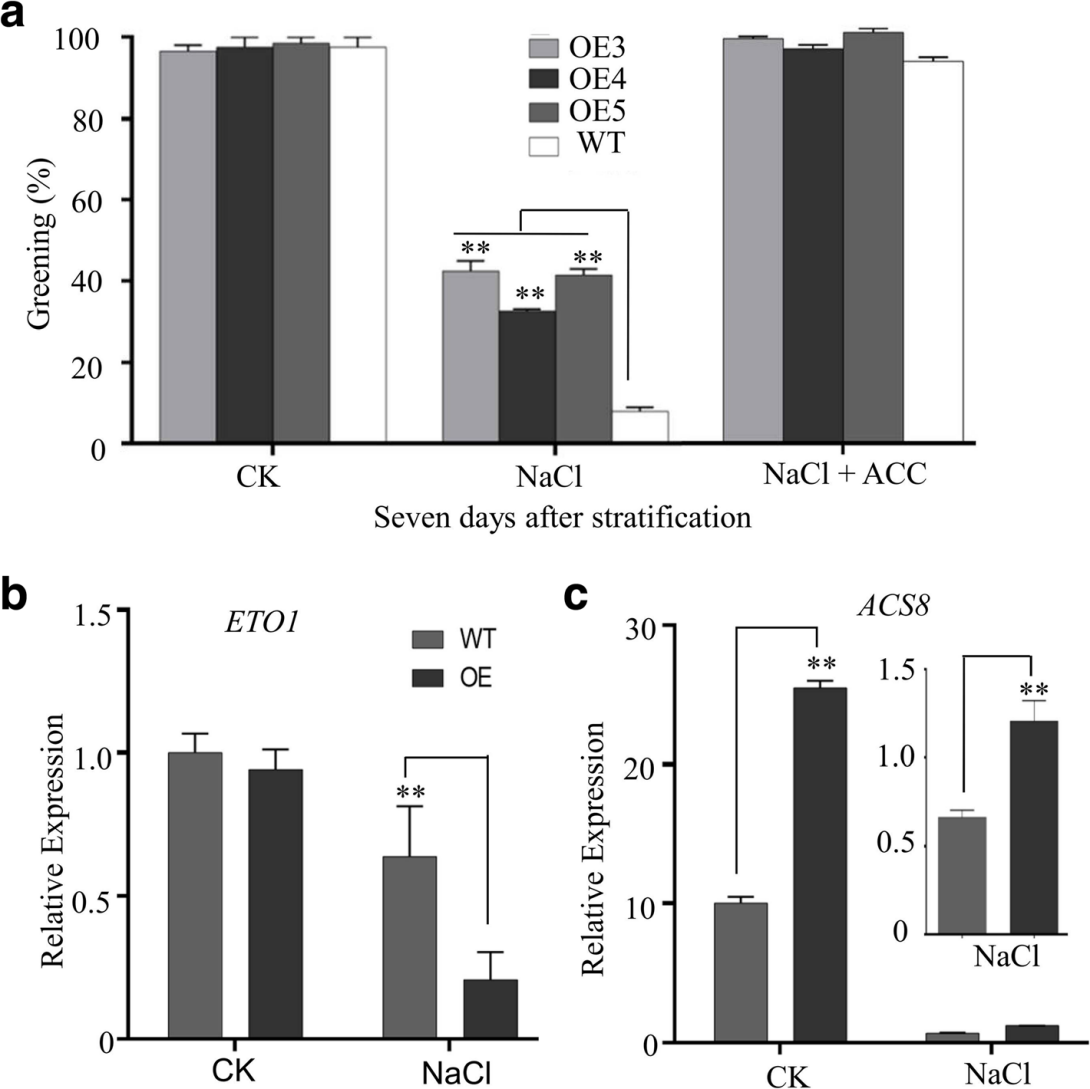
Responses of GhPLATZ1 transgenic and WT plants to ethylene. **a** Cotyledon greening of WT, OE3, OE4, and OE5 seedlings grown on 1/2 MS medium with or without 175 mM NaCl, or 175 mM NaCl and 5 μM ACC for 14 days. Data show the mean ± SD (*n* = 49) of three replicates. **b** The relative expression of *ETO1* and **c** *ACS8* in 3.5-d germinated seeds of WT and OE3 on 1/2 MS medium with or without 175 mM NaCl. At least three independent experiments were conducted

**Fig. 7:**
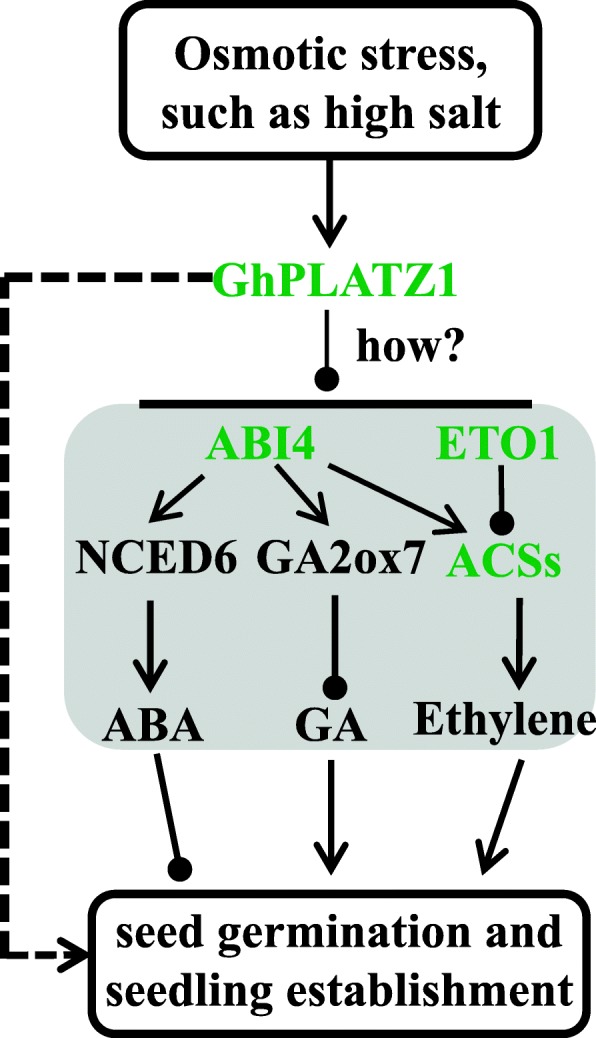
Model of GhPLATZ1 transgenci Arobidopsis seedlings responses to osmotic stresses. Words in green and black colors indicates determined gene expression in this study and previous reported results, respectively

We also found that seed germination was less sensitive to NaCl after the addition of exogenous ACC in both *GhPLATZ1* transgenic lines and WT (Fig. 6a). *ETO1* (Ethylene-overproduction protein 1) was significantly down-regulated by twofold under salt stress in *GhPLATZ1* transgenic lines compared with WT (Fig. 7b). *ACS8* was up-regulated by 1.5 times under control and salt stress conditions in *GhPLATZ1* transgenic lines compared with WT (Fig. 7c). Thus, GhPLATZ1 was involved in the ethylene-mediated salt stress response in plants during seed germination and postgermination growth.

## Discussion

More than 10 years ago, PLATZ1 was isolated from pea and characterized as a plant-specific zinc-dependent transcription repressor [13]. Recently, only Arabidopsis PLATZ1 and 2 [19], and maize PLATZ protein FL3 [20] were reported to be involved in abiotic stress and seed development, respectively. Therefore, the roles of PLATZs remain largely unclear.

In the present study, we illustrated that the *GhPLATZ1* expression was significantly induced by salt, mannitol, ABA and GA in 20-day-old plants and germinated seeds (Fig. 1a and b), thereby suggesting the involvement of GhPLATZ1 in multiple abiotic stress responses. *GhPLATZ1* transgenic lines showed significantly enhanced osmotic, salt, ABA, and PAC insensitivity during seed germination and seedling establishment (Figs. 2, 3, 4 and 5). This function of GhPLATZ1 is similar to that of AtPLATZ1 and AtPLATZ2 whose mutants *platz1* and *platz2* increased sensitivity to ABA [19]; thus GhPLATZ1 might function similarly to AtPLATZ1 and AtLATZ2 during seed germination. However, *GhPLATZ1* transgenic plants showed similar phenotypes to WT in vegetative tissues under normal, salt and mannitol conditions (data not shown), whereas constitutive expression of AtPLATZ1 conferred tolerance to low water availability [19]. Therefore, PLATZs from different species may function similarly in some developing stages but differently in other developing stages in response to abiotic stresses.

Impaired germination and postgermination growth are common among seeds exposed to salt and drought stresses. Plants can regulate and coordinate both growth and/or stress tolerance to promote survival or escape from environmental stress by modifying the production, distribution, or signal transduction of hormones. The hormonal balance between ABA and GA in seed germination in response to environmental stimuli is well documented and discussed [5, 22–24]. ABA plays crucial roles in dormancy induction in developing seeds and in dormancy maintenance in seed imbibition, whereas GAs are involved in germination. Exogenous ABA application inhibits germination in a dose-dependent manner. Applying fluridone, an ABA biosynthesis inhibitor, substantially stimulates seed germination under salinity [1]. The seeds of typical ABA-deficient mutants germinate faster than those of WT [25], and transgenic plants constitutively expressing the ABA biosynthesis gene maintain considerable seed dormancy [26, 27]. In contrast, GA_3_ enhances germination under NaCl stress [28]. GA-deficient mutants, such as *ga1* and *ga2*, show strong seed dormancy and fail to germinate without exogenous GA treatment [2]. Mutants that are defective in GA 2-oxidases, which deactivate bioactive GA, show decreased seed dormancy [29]. Compared with WT, our data indicated low levels of ABA in dry seeds of GhPLATZ1 transgenic *Arabidopsis* (Fig. 4a). This result may lead to the insensitivity of GhPLATZ1 transgenic *Arabidopsis* seeds to osmotic stress and ABA compared with WT. The down-regulation of *ABI4* expression (Fig. 4c) confirmed the insensitivity of GhPLATZ1 transgenic *Arabidopsis* seeds to osmotic stress and ABA. By contrast, the different responses of GhPLATZ1 transgenic and WT *Arabidopsis* to PAC indicated the potential high GA in GhPLATZ1 transgenic *Arabidopsis* in response to salt stress. Therefore, the major active GA forms such as GA_1_ and GA_4_ need further investigated. The transcription of a key ABA biosynthetic gene, *NCED6*, and key GA catabolic gene, *GA2ox7*, is significantly enhanced by *ABI4* overexpression by directly binding to their promoters; and ABA induces *GA2ox7* transcription whereas GA represses *NCED6* expression in an ABI4-dependent manner; and ABA stabilizes the ABI4 protein whereas GA promotes its degradation [30]. Thus, GhPLATZ1 might function in ABA and GA antagonism by suppressing *ABI4* transcription and inhibiting the activating expression of *NCED6* and *GA2ox7*, thereby leading to decreased ABA and increased GA (Fig. 7).

In addition, ethylene accelerates seed germination by overcoming the inhibitory action of ABA or stimulating GA biosynthesis or its signaling pathway [31]. Two results indicated that GhPLATZ1 increased ethylene production: first, *ACS8*, a gene that encodes ethylene biosynthesis, showed up-regulated expression in *GhPLATZ1* transgenic lines compared with WT (Fig. 6c). *ABI4* transcripts were significantly suppressed in *GhPLATZ1* transgenic lines (Fig. 4c). Increasing evidence has revealed that ABI4 binds directly to promoters of *ACS4*, *ACS8*, and *ACO2* to inhibit their transcription causing reduced ethylene production [32]. Therefore, significantly suppressed *ABI4* transcripts in *GhPLATZ1* transgenic lines suggested the up-regulated expression of ethylene biosynthesis genes, such as *ACS4*, *ACS8*, and *ACO2*. Here, we detected the expression of ACS8, a member of a gene family encoding ethylene biosynthetic enzymes, was up-regulated in GhPLATZ1 in transgenic lines (Fig. 6c). Second, *ETO1* showed significantly down-regulated expression in *GhPLATZ1* transgenic lines (Fig. 6b). ETO1 is a component of the E3-ligase complex and interacts directly with ACS5 and Le-ACS3 for degradation in a proteasome-dependent manner [32–35]. Mutated ETO1 forms result in increased ACS5 protein stability and ethylene overproduction [33]. Therefore, the significantly down-regulation of *ETO1* in *GhPLATZ1* transgenic lines (Fig. 6b) also suggested increased ethylene levels in *GhPLATZ1* transgenic lines. The results of ACC (the direct precursor of ethylene) application (Fig. 7a) further indicated the higher ethylene in *GhPLATZ1* transgenic lines than WT. Thus, GhPLATZ1 might increase ethylene production by down-regulating the expression of *ABI4* and *ETO1* genes (Fig. 7).

## Conclusions

In conclusion, this study discovered a new regulator, *GhPLATZ1*, which was induced by NaCl, mannitol, ABA and GA treatments. The model in Fig. 7 shows how *GhPLATZ1* functions in osmotic stresses during seed germination and seedling establishment. First, GhPLATZ1 increases osmotic insensitivity of transgenic *Arabidopsis* by inhibiting *ABI4* expression, which can induce the expression of *NCED6* and *GA2ox7*. Second, GhPLATZ1 inhibits *ABI4* and *ETO1* expression, which can suppress *ACS* gene expression and decrease ACS protein stability, respectively. Therefore, GA and ethylene possibly increased and ABA decreases in *GhPLATZ1* transgenic Arabidopsis to promote seed germination and seedling establishment under osmotic stress. However, overexpression of ABI4 and ETO1 in GhPATZ1 transgenic Arabidopsis will confirm this conclusion. However, GhPLATZ1 regulates the expression levels of *ABI4* and *ETO1*, as well as what and how other signaling participate in GhPLATZ1-mediated pathways remains unknown.

## Methods

### Plant materials and treatments

Seeds of upland cotton (*Gossypium hirtusium.L*) cultivar ZM19 were bought from Chinese Academy of Agricultural Sciences. Seedlings were grown for 20 days in MS liquid medium in a growth chamber with 300 μM m^− 2^·s^− 1^ light intensity and day/night temperatures of 28 °C/20 °C. For each treatment, every 15 uniformly developed seedlings were transferred to MS media containing either 200 mM NaCl, 300 mM mannitol, 50 μM ABA or 50 μM GA_3_ for 12 h. In addition, seeds germinated in water for 2.5 days with 0.5 cm radicles were also treated with MS media containing either 200 mM NaCl, 300 mM mannitol, 50 μM ABA or 50 μM GA_3_ for 6 and 12 h, respectively. Twenty-day seedlings or 2.5-d germinated seeds transferred to MS media were used as control. Samples were harvested at the indicated points, frozen in liquid nitrogen, and stored at − 70 °C for RNA extraction. Each treatment was repeated three times.

### Vector construction and transformation

To construct 35S::*GhPLATZ1*, the *GhPLATZ1* coding sequence was amplified using cDNA by PCR with gene specific primers (Additional file 2: Table S1). The resulting PCR product was cloned into the *Sal*I and *Kpn*I sites of binary vector pBI121 under the control of a cauliflower mosaic virus 35S promoter.

The 35S::*GhPLATZ1* was then introduced into *Agrobacterium tumefaciens* strain GV3101 to be transformed into *Arabidopsis thaliana* ecotype Columbia-0 by floral dipping [36]. The transgenic plants were screened on 1/2 MS medium containing 50 mg·L^− 1^. kanamycin. Two generations of the corresponding T_1_ transgenic seedlings segregated at a ratio of 3:1 (resistant:sensitive) were selected to propagate T_3_ homozygous. Some of the transgenic *Arabidopsis* plants were confirmed by detecting the expression of *GhPLATZ1* by PCR and used for further experiments. Primers are listed in Additional file 2: Table S1.

### RNA extraction

The total RNAs of different cotton samples used in this report were isolated using RNeasy Plant Mini Kit (QIAGEN). The total RNAs of 20-day-old seedlings and germinated seeds of different *Arabidopsis* transgenic lines were isolated using a Universal Plant Total RNA Extraction Kit (spin-column)-I (BioTeke Beijing China).

### Quantitative RT-PCR

qRT-PCR was performed as previously described [37]. Cotton *GhUBI* (EU604080) or *Arabidopsis actin2* genes were used as the standard control. The relative *GhPLATZ1 and AtPLATZs* expression level was analyzed using the comparative CT method, and three replicates of each sample were analyzed. All of the primers used are listed in Additional file 2: Table S1. At least three independent experiments were carried out.

### Fluorescence microscopy

Roots from seven-day-old transgenic Arabidopsis containing 35S::*GhPL-GFP* were used and imaged by LSCM51 (Zeiss) at 488 nm for GFP.

### Seed germination and cotyledon greening analysis

Seeds of each genotype were harvested from plants of the same age and stored for 4 weeks in the dark at 4 °C. For each comparison, seeds were surface-sterilized with 70% ethanol for 5 min, subsequently incubated in 2.6% sodium hypochlorite for 10 min, and washed five times with sterile water. At least 30 sterile seeds were plated on 1/2 MS medium plus 1% (*w*/*v*) sucrose with or without different concentrations of NaCl, KCl, Na_2_SO_4_, LiCl, mannitol, ABA, PAC, or NaCl combined with GA or ACC as indicated. Plates were chilled at 4 °C in the dark for 3 days (stratification) and moved to 22 °C with a 16-h-light/8-h-dark cycle. The percentage of seed germination was determined at indicated time points. Germination was defined as an obvious emergence of the radicle through the seed coat. The percentage of cotyledon greening was recorded at 14 days after the end of stratification. Cotyledon greening was defined as obvious cotyledon expansion and turning of cotyledon color into green. Three biological replications were performed.

### ABA content measurements

To quantify ABA content, dry seeds and two-day germinated seeds of GhPLATZ1 overexpressing *Arabidopsis* and WT treated with or without 175 mM NaCl were ground in liquid nitrogen. Furthermore, 150 mg of seed powder was homogenized and extracted for 24 h in methanol containing D6-ABA (OIChemIm Co., Ltd.) as an internal standard. The mixture was purified with an Oasis Max solid-phase extract cartridge (150 mg/6 cc; waters) and eluted with 5% formic acid in methanol. The elution was dried and reconstituted. ABA content was measured by HPLC-MS-MS (Agilent 1290 Rapid-resolution Liquid chromatography, Agilent Technologies, Waldbronn, Germany; Sciex 6500 Q-Trap AB Technologies, USA). The mobile phase consisted of A:B (methyl alcohol/0.1% methanoic acid:H_2_O/0.1% methanoic acid). The elution gradient was 0–2 min, A = 20%; 2–14 min, A = 80%; and 15.1–20 min, A = 20%. The injection volume was 2 μL. The mass spectrometer conditions were as follows: spray voltage, 4500 V; atomizer pressure, 65 psi; assist device pressure, 70 psi; atomization temperature, 400 °C.

### Statistical analysis

Data were subjected to data processing system and significant differences were established through one-way ANOVA. Differences at 5% and 1% levels were considered significant and denoted by single and double stars (*P < 0.05; **P < 0.01).

## Additional files

Additional file 1: **Figure S1.** Sequence analysis of GhPLATZ1 (GenBank accession no. AFH57272) with its homologs. (**A**) Multiple alignments of GhPLATZs. Identical amino acids are shaded in black. The conserved zinc-fingers are indicated by C and H at the bottom of the sequences. (**B**) Phylogenetic analysis of GhPLATZs and AtPLATZs. The GenBank accession numbers are as follows: GhPLATZ1 (XP_016742770.1), GhPLATZ2 (XP_016736692.1), GhPLATZ3 (XP_016705363.1), GhPLATZ4 (XP_01672128.1), GhPLATZ5 (XP_012488860.1), GhPLATZ6 (XP_016670611.1), GhPLATZ7 (XP_016723269.1), GhPLATZ8 (XP_016728634.1), GhPLATZ9 (XP_016742383.1), AtPLATZ1.1 (AT1G21000.1), AtPLATZ1.2 (AT1G21000.2), AtPLATZ2 (AT1G76590.1), AtPLATZ3.1 (AT1G32700.1), AtPLATZ3.2 (AT1G32700.2), AtPLATZ4 (AT1G43000.1), AtPLATZ5 (AT1G31040.1), AtPLATZ6 (AT2G01818.1), AtPLATZ7 (AT2G12646.1), AtPLATZ8 (AT2G27930.1), AtPLATZ9 (AT3G50808.1), AtPLATZ10 (AT3G60670.1), AtPLATZ11.1 (AT4G17900.1), AtPLATZ11.2 (T4G17900.2), AtPLATZ12 (AT5G46710.1). **Figure S2.** Phenotypes of *GhPLATZ1* transgenic Arabidopsis under salt conditions. **(A)** Photographs taken at 3, 5, and 7 d on 1/2 MS medium containing different concentrations of NaCl. **(B)** Germination rates of WT and OE-3 seeds on 1/2 MS medium with or without 175 or 200 mM NaCl in (A). **(C)** Cotyledon greening of WT and OE-3 seedlings grown on 1/2 MS medium with or without 150, 175 or 200 mM NaCl in (A). **Figure S3.** Phenotypic analysis of WT and *GhPLATZ1* transgenic Arabidopsis seeds on 1/2 MS medium for 3.5 d (A) and 1/2 MS medium with or without 200 mM NaCl for14 d (B). **Figure S4.** Phenotypes of *GhPLATZ1* transgenic Arabidopsis under potassium, sodium and lithium stresses. Germination rates of WT, OE-3, OE-4, and OE-5 seeds on 1/2 MS medium with or without 175 mM KCl (**A**), 100 mM Na_2_SO_4_ (**B**), and 15 mM LiCl (**C**) for indicated times. (**D**) Cotyledon-greening of WT, OE-3, OE-4, and OE-5 seedlings grown on 1/2 MS medium with or without 15 mM LiCl for 14 days. **Figure S5.** Expression of *AtPLATZ* genes in GhPLATZ1 transgenic and WT plants. AtPLATZ1 (AT1G210001), AtPLATZ2 (AT1G76590.1), AtPLATZ3.1 (AT1G32700.1), AtPLATZ4 (AT1G43000.1), AtPLATZ5 (AT1G31040.1), AtPLATZ6 (AT2G01818.1), AtPLATZ7 (AT2G12646.1), AtPLATZ8 (AT2G27930.1), AtPLATZ9 (AT3G50808.1), AtPLATZ10 (AT3G60670.1), AtPLATZ11.1 (AT4G17900.1), AtPLATZ12 (AT5G46710.1). Primers used here are listed in Additional file 2: Table S1. (DOCX 21 kb)

Additional file 2: **Table S1.** List of primers used in this study. (DOCX 21 kb)

## Abbreviations

ABA: Abscisic acid
ABI: ABA insensitive
ACC: 1-aminocyclopropane-1-carboxylic acid
ACS: ACC synthase
CK: Control
ETO1: Ethylene-overproduction protein 1
GA: Gibberellin
MS: Murashige and Skoog
OE: Transgenic arabidopsis with high *GhPLATZ1* overexpression
PAC: Paclobutrazol
WT: Wild type
