# Supplementary figures and images for: Expression of cotton PLATZ1 in transgenic Arabidopsis reduces sensitivity to osmotic and salt stress for germination and seedling establishment associated with modification of the abscisic acid, gibberellin, and ethylene signalling pathways
Source: BMC Plant Biol. 2018 Oct 4;18:218. doi: 10.1186/s12870-018-1416-0 (PMC6172764; doi:10.1186/s12870-018-1416-0)

Additional file 1

**Figure. S1**


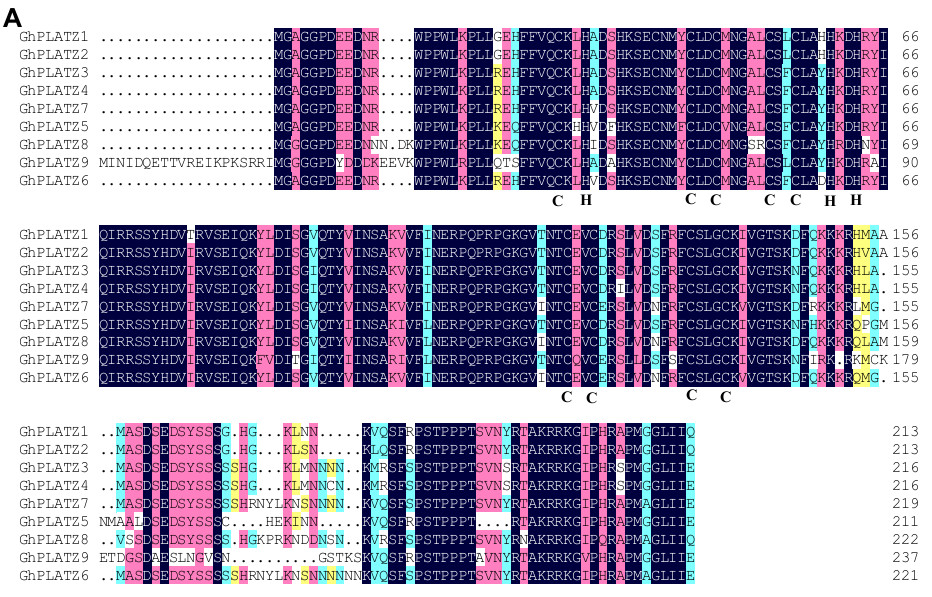


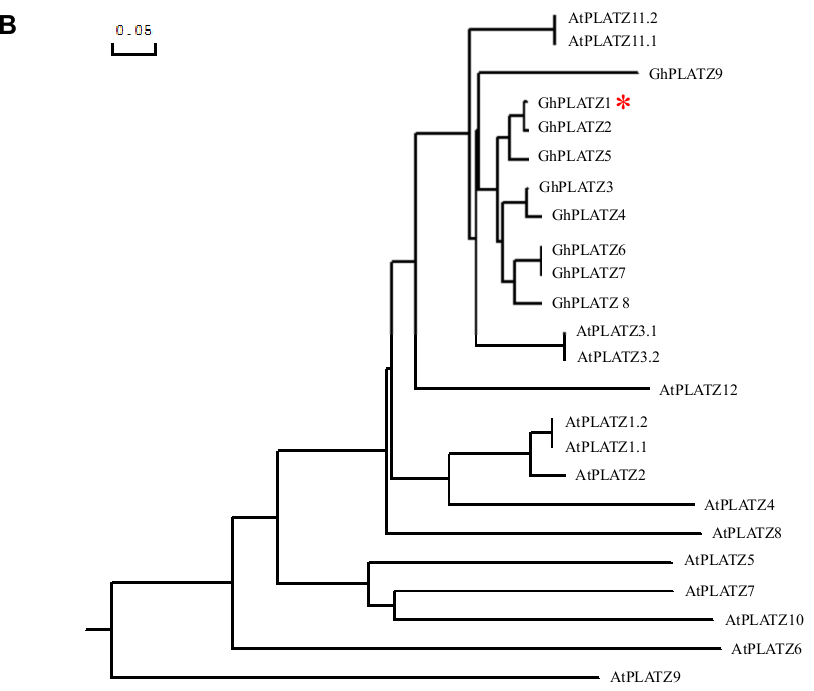


**Figure. S2**


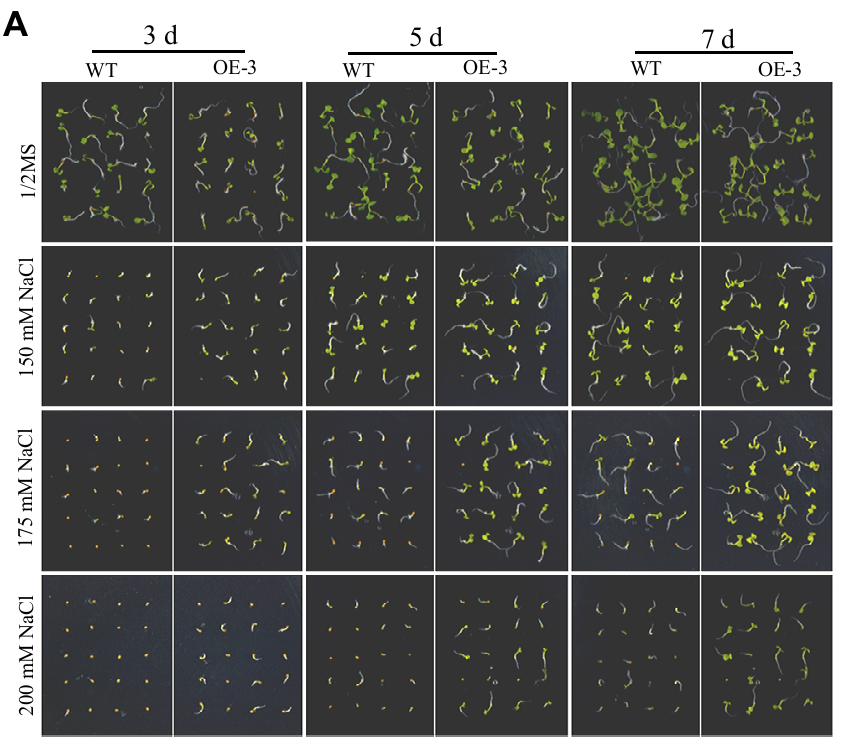

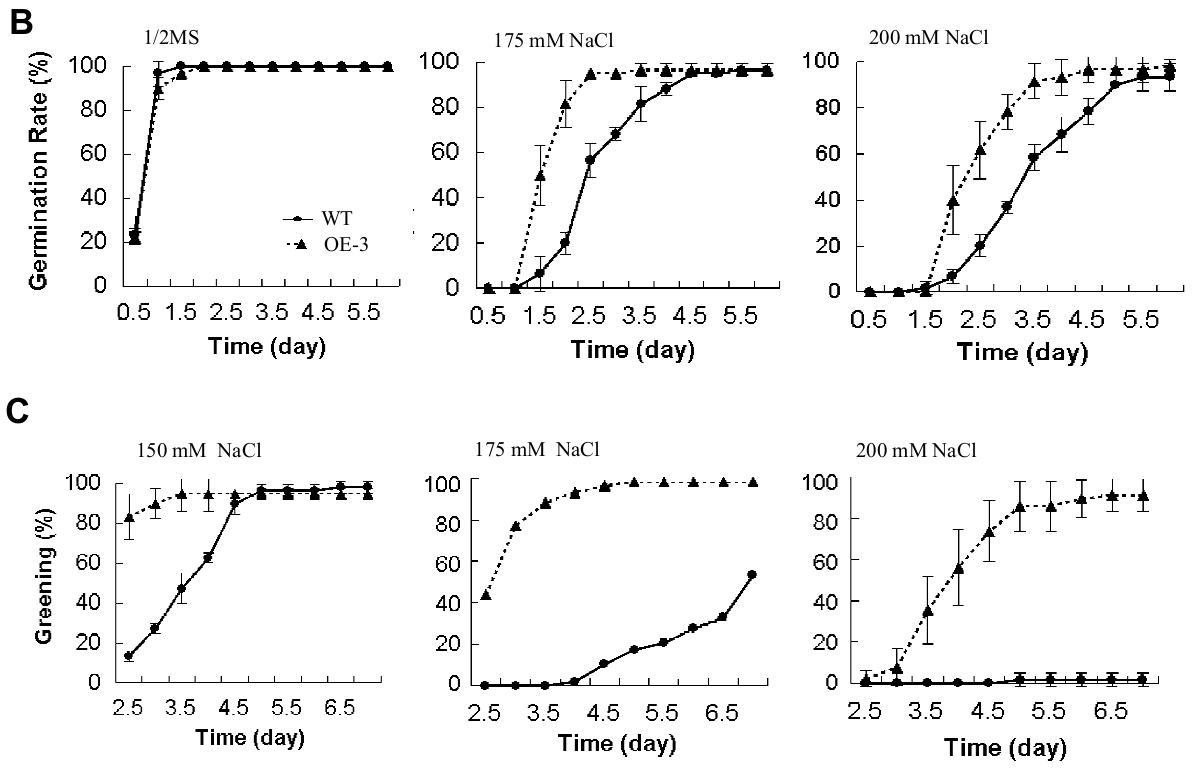


**Figure S3**


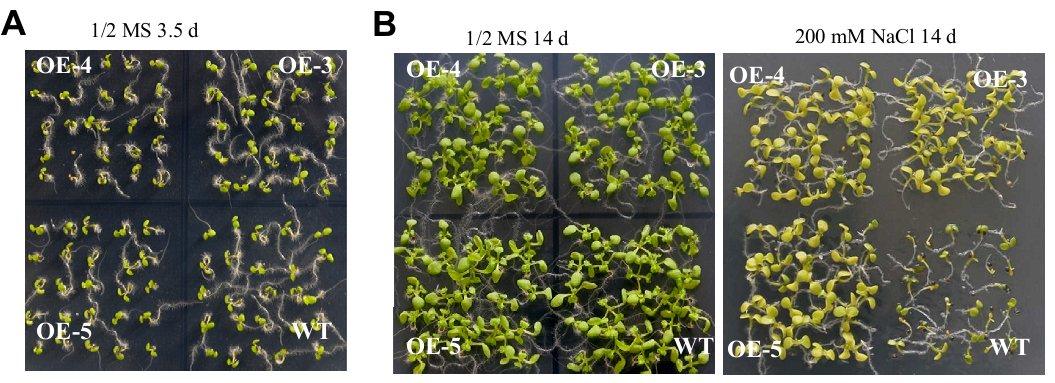


**Figure. S4**


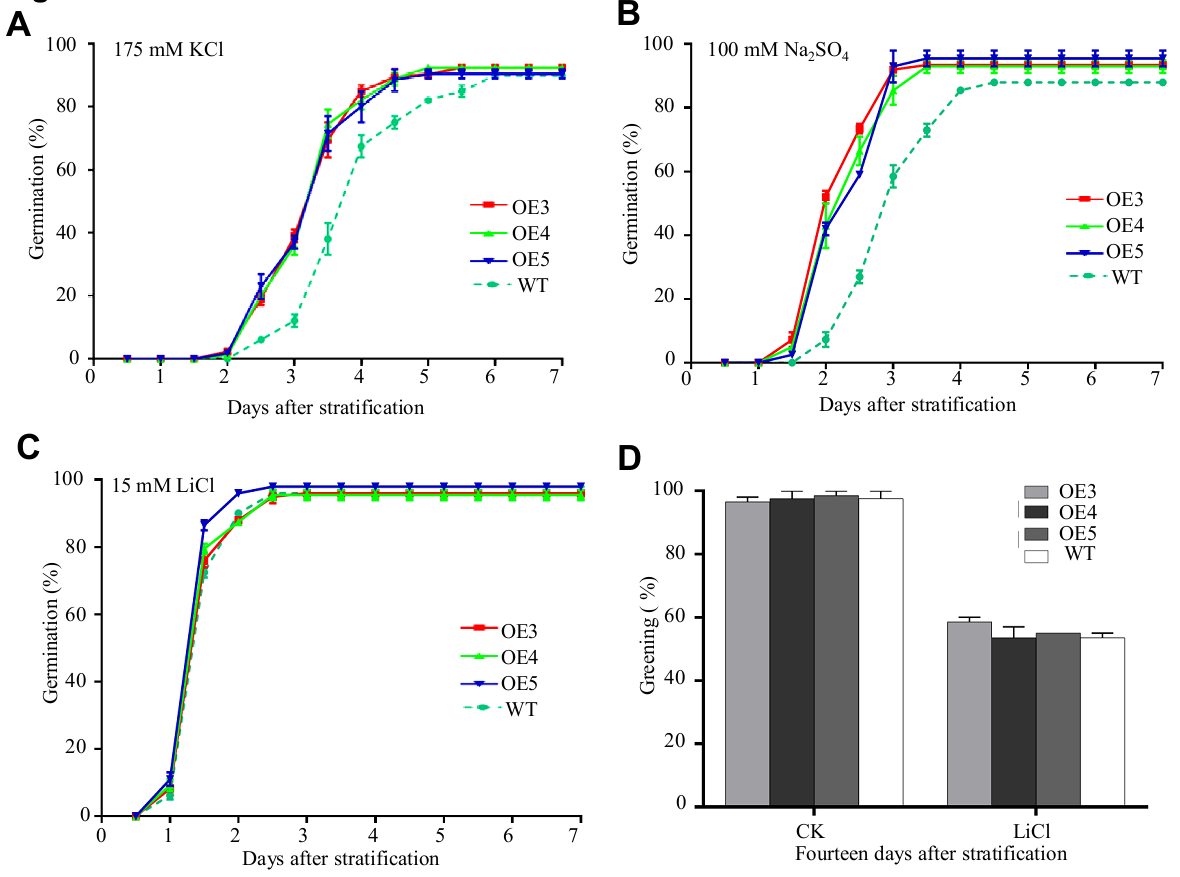


**Figure. S5**


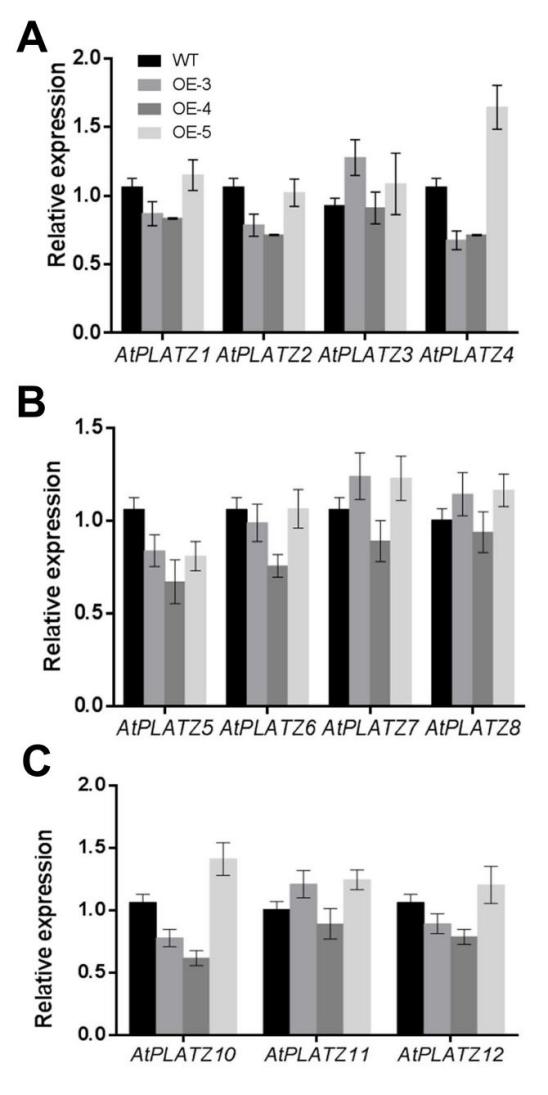

Supplement: Supplementary file 1 — Figure S1. Sequence analysis of GhPLATZ1 (GenBank accession no. AFH57272) with its homologs. (A) Multiple alignments of GhPLATZs. Identical amino acids are shaded in black. The conserved zinc-fingers are indicated by C and H at the bottom of the sequences. (B) Phylogenetic analysis of GhPLATZs and AtPLATZs. The GenBank accession numbers are as follows: GhPLATZ1 (XP_016742770.1), GhPLATZ2 (XP_016736692.1), GhPLATZ3 (XP_016705363.1), GhPLATZ4 (XP_01672128.1), GhPLATZ5 (XP_012488860.1), GhPLATZ6 (XP_016670611.1), GhPLATZ7 (XP_016723269.1), GhPLATZ8 (XP_016728634.1), GhPLATZ9 (XP_016742383.1), AtPLATZ1.1 (AT1G21000.1), AtPLATZ1.2 (AT1G21000.2), AtPLATZ2 (AT1G76590.1), AtPLATZ3.1 (AT1G32700.1), AtPLATZ3.2 (AT1G32700.2), AtPLATZ4 (AT1G43000.1), AtPLATZ5 (AT1G31040.1), AtPLATZ6 (AT2G01818.1), AtPLATZ7 (AT2G12646.1), AtPLATZ8 (AT2G27930.1), AtPLATZ9 (AT3G50808.1), AtPLATZ10 (AT3G60670.1), AtPLATZ11.1 (AT4G17900.1), AtPLATZ11.2 (T4G17900.2), AtPLATZ12 (AT5G46710.1). Figure S2. Phenotypes of GhPLATZ1 transgenic Arabidopsis under salt conditions. (A) Photographs taken at 3, 5, and 7 d on 1/2 MS medium containing different concentrations of NaCl. (B) Germination rates of WT and OE-3 seeds on 1/2 MS medium with or without 175 or 200 mM NaCl in (A). (C) Cotyledon greening of WT and OE-3 seedlings grown on 1/2 MS medium with or without 150, 175 or 200 mM NaCl in (A). Figure S3. Phenotypic analysis of WT and GhPLATZ1 transgenic Arabidopsis seeds on 1/2 MS medium for 3.5 d (A) and 1/2 MS medium with or without 200 mM NaCl for14 d (B). Figure S4. Phenotypes of GhPLATZ1 transgenic Arabidopsis under potassium, sodium and lithium stresses. Germination rates of WT, OE-3, OE-4, and OE-5 seeds on 1/2 MS medium with or without 175 mM KCl (A), 100 mM Na2SO4 (B), and 15 mM LiCl (C) for indicated times. (D) Cotyledon-greening of WT, OE-3, OE-4, and OE-5 seedlings grown on 1/2 MS medium with or without 15 mM LiCl for 14 days. Figure S5. Expression of AtPLATZ genes in GhPLATZ1 transgenic and WT [file 12870_2018_1416_MOESM1_ESM.docx]
