# Supplementary material for: Expression of cotton PLATZ1 in transgenic Arabidopsis reduces sensitivity to osmotic and salt stress for germination and seedling establishment associated with modification of the abscisic acid, gibberellin, and ethylene signalling pathways
Source: BMC Plant Biol. 2018 Oct 4;18:218. doi: 10.1186/s12870-018-1416-0 (PMC6172764; doi:10.1186/s12870-018-1416-0)
Supplement: Supplementary file 2 — Table S1. List of primers used in this study. (DOCX 21 kb) [file 12870_2018_1416_MOESM2_ESM.docx]

**Additional file 2**

**Table. S1** List of primers used in this study.

| PLATZ qRT-F | GGAGGTCCTCATACCATGATGTG |
| --- | --- |
| PLATZ qRT-R | CTACGGTCACAGACCTCACAGG |
| PLATZ RT-F | ATGGATTACAAGGATGATGATGATAAG |
| PLATZ RT-R | TTAATATTGTATGATTAGTCCTCC |
| GhUBI-F | TGCTGGGAAACAACTGGAAG |
| GhUBI-R | GAAGACGAAGAACAAGGTGAAG |
| PLATZ-F | TCTAGAATGGGTGCTGGTGGAC |
| PLATZ-R | GGATCCATATTGTATGATTAGTCCTCCCA |
| GhPLATZ-GFP-F | TCTAGAATGGGTGCTGGTGGAC |
| GhPLATZ-GFP-R | GGTACCATATTGTATGATTAGTCCTCCCA |
| PLATZ-LUC-F | GGTACCATGGGTGCTGGTGGAC |
| PLATZ-LUC-R | GGATCCATATTGTATGATTAGTCCTCCCA |
| PLATZ-N-F | GGTACCATGGGTGCTGGTGGAC |
| PLATZ-N-R | GGATCCCACAACCTTAGCGCTGTT |
| PLATZ-C-F | GGTACCAACAGCGCTAAGGTTGTG |
| PLATZ-C-R | GGATCCATATTGTATGATTAGTCCTCCCA |
| ABI4-F | CTCTTCCTCCTCCGTCTC |
| ABI4-R | GTTGGCTCCTCCTCCTAC |
| ABI5-F | AACCTAATCCAACCCGAACC |
| ABI5-R | ACCCTCCTCCTCCTGTCC |
| ETO1-F | TCCGAACCTATCCTTACA |
| ETO1-R | CATAATGCTGCTTCACAAT |
| ACS8-F | TGATTTACTCCAACGATGATTT |
| ACS8-R | GGTGAAGGTCTTGTCTGA |
| JAZ9-F | GAATCCATGGAAAGAGATTTTCTGGG |
| JAZ9-R | CTGCAGTGTAGGAGAAGTAGAAGAGTAATTC |
| AtPLATZ1-F | CTTGGAGGAATGAGGAGAG |
| AtPLATZ1-R | ATCTTAGTCGGTGTAGTAGC |
| AtPLATZ2-F | TCCTTCAAGAACCATTCCA |
| AtPLATZ2-R | ATGTCCGTCCATTCTCAA |
| AtPLATZ3-F | TCGGATTCTGATGATTCGTA |
| AtPLATZ3-R | CTGATAAAGGCGGTGTTG |
| AtPLATZ4-F | ATCTGTTCTCGTAACCTTCT |
| AtPLATZ4-R | GTCATCACTCTTTCCACTATG |
| AtPLATZ5-F | CGTCTGATGAGTGGTCTT |
| AtPLATZ5-R | CCCTTCCGTCTACTTGAA |
| AtPLATZ6-F | GCTTCATGTGTGACTTGTAA |
| AtPLATZ6-R | TTCCTTCTTGAGTAGAGTGTT |
| AtPLATZ7-F | GCCACACTCTAACCCTAG |
| AtPLATZ7-R | CCGTCTACCACAGTTGAT |
| AtPLATZ8-F | GAAGGAATGAGGAAGAATAAGG |
| AtPLATZ8-R | AGAATTAGAGGTATGAGTTGGA |
| AtPLATZ10-F | TCCGAGTCAAGTGGTAAC |
| AtPLATZ10-R | TCCGTGTTAGTCGTTGAA |
| AtPLATZ11-F | AAGTCGTCTTCCTGAATGA |
| AtPLATZ11-R | AAGCCTCTTGATGTTCCA |
| AtPLATZ12-F | GAGTGAAGCATACAACGAAT |
| AtPLATZ12-R | GTGTAGGTGGACTGAGAC |
| Actin2-F | CTTCGTCTTCCACTTCAG |
| Actin2-R | ATCATACCAGTCTCAACAC |
